# Characterizing trimodal therapy outcomes by HIV status in early-stage cervical cancer: a retrospective cohort study from a Kenyan tertiary centre

**DOI:** 10.1186/s12885-026-15761-5

**Published:** 2026-03-09

**Authors:** Gabriel Eliazaro Ouma, Kimbley Asaso Omwodo, Peter Itsura, Philippe Amubuomombe Poli, Kanguru Wahome, Odhiambo Otieno, Nasengo Chiriswa, Green Harris Jose, Adagi Awuor, Barry Rosen, Allan Covens, Philip Tonui

**Affiliations:** 1 Department of Reproductive Health, Moi University, Eldoret, Kenya; 2 Obstetrics and Gynecology, The Nairobi Hospital, Nairobi, Kenya; 3 Moi Teaching and Referral Hospital, Eldoret, Kenya; 4 Jaramogi Oginga Odinga Teaching and Referral Hospital, Kisumu, Kenya; 5 University of Michigan, Michigan, AL United States of America; 6 University of Toronto, Toronto, Canada

**Keywords:** Cervical cancer, Radical Hysterectomy, Concurrent chemoradiotherapy, Trimodal therapy, Human Immunodeficiency Virus (HIV), Kenya

## Abstract

**Background:**

Adjuvant concurrent chemoradiotherapy following radical hysterectomy is the cornerstone of curative-intent treatment for early-stage cervical cancer. However, among people living with Human Immunodeficiency Virus (PLWHIV) and cervical cancer, the interplay of treatment toxicity, immunosuppression and systemic health barriers presents a compounded clinical challenge. Evidence on how HIV infection influences baseline clinical patterns and prognosis following standard multimodal therapy remains poorly characterized.

**Methods:**

We conducted a descriptive retrospective cohort review of women with FIGO 2018 stage IA–IIA cervical cancer who had completed curative-intent trimodal therapy (adjuvant pelvic external-beam radiotherapy (45–50.4 Gy), weekly cisplatin (40 mg/m²), and brachytherapy following radical hysterectomy (type II/III) with pelvic lymphadenectomy) between 2014 and 2023, at a tertiary referral hospital in Kenya. The baseline clinicopathological characteristics, treatment-related toxicities, 3-year disease-free survival (DFS), and 5-year overall survival (OS) were described. Results were stratified by HIV status. Survival analysis was conducted using Kaplan-Meier estimates and log-rank tests.

**Results:**

Over the 10-year study period, 275 patients with cervical cancer underwent radical hysterectomy with bilateral pelvic lymphadenectomy. Of 62 patients meeting criteria for adjuvant therapy (17 PLWHIV, 45 HIV-negative), 38 (61.3%) completed trimodal therapy. This corresponded to a completion rate of 76.5% (13/17) among PLWHIV versus 55.6% (25/45) among HIV-negative patients. Baseline clinicopathological profiles, including age, performance status, and histology (squamous cell carcinoma: 100% versus 88.0%) did not differ substantially between groups. Positive lymph nodes were the most common high-risk feature (53.8% versus 40.0%), and lymphovascular space invasion (LVSI) was the predominant intermediate-risk feature (69.2% versus 40.0%). Median time from surgery to adjuvant therapy initiation was 77.5 days (IQR 42–210). Lymphedema (21.1%) and bladder dysfunction (18.4%) were the frequently reported any-grade chronic toxicities. Myelosuppression occurred in 23.1% versus 4.0%. The 3-year DFS was 53.8% among PLWHIV and 77.6% among HIV-negative patients (log-rank *p* = 0.14); median OS was 14.5 months versus 21.1 months (log-rank *p* = 0.12). Five-year survival estimates were not attainable for PLWHIV due to early recurrence and mortality.

**Conclusion:**

Despite comparable baseline characteristics, PLWHIV showed a nonsignificant trend toward greater treatment-related toxicity and reduced survival following trimodal therapy for early-stage cervical cancer. These findings underscore the importance of regional strengthening of HIV-oncology integrated services, and generating prospective research on optimum management strategies for this vulnerable cohort.

**Supplementary Information:**

The online version contains supplementary material available at 10.1186/s12885-026-15761-5.

## Key Message

While concurrent chemoradiotherapy following radical hysterectomy is standard in management of early-stage cervical cancer with intermediate/high-risk features, our findings highlight that treatment completion rates are suboptimal. Moreover, the median time from surgery to adjuvant therapy initiation exceeds the recommended 6-week period. PLWHIV experienced numerically higher treatment related morbidity (especially myelosuppression), and reduced 3-year DFS compared to their HIV-negative counterparts.

There is a need for heightened vigilance and proactive management of myelosuppression among PLWHIV undergoing trimodal therapy. At systems level, survival gains may require addressing systemic barriers, specifically reducing delays in adjuvant therapy. Further research is required to explore mechanisms driving outcome disparities and to test integrated supportive care interventions in order to achieve optimum management for this special population.

**Supplementary Information:**

The online version contains supplementary material available at 10.1186/s12885-026-15761-5.

## Introduction

Cervical cancer is the fourth most common cancer among women worldwide, with nearly 90% of related deaths occurring in low- and middle-income countries (LMICs) [1, 2]. People living with HIV (PLWHIV) have a sixfold higher risk of developing cervical cancer compared to the general population, and approximately 5% of all cervical cancer cases are attributable to HIV infection [3]. As an AIDS-defining malignancy, cervical cancer in PLWHIV presents unique challenges in management due to the interplay of immunosuppression, adverse drug interactions, increased susceptibility to opportunistic infections, and psychosocial vulnerabilities [4, 5].

The introduction of antiretroviral therapy (ART) has led to marked improvements in immune function and overall health outcomes for PLWHIV. For early-stage cervical cancer, treatment options include radical surgery with tailored adjuvant therapy or primary concurrent chemoradiotherapy (CCRT). No single approach has demonstrated superiority in terms of overall survival. However, patients with FIGO 2018 stage IB–IIA disease who underwent radical surgery experienced higher rates of severe morbidity compared to those who received primary radiotherapy, while combined surgery and radiotherapy resulted in the greatest treatment-related morbidity [6]. In LMICs where preoperative staging is limited and surgical pathology more often guides adjuvant decisions, trimodal therapy remains preferred. Additionally, brachytherapy access is frequently constrained to post-surgical patients, making the trimodal regimen a pragmatic standard of care.

In Kenya, a prior study at our institution suggested poorer overall survival among PLWHIV compared to their HIV-negative counterparts following radical hysterectomy; causality was however not established, and trimodal regimen was not evaluated [7]. Other studies demonstrated that, for stage IIB disease, CCRT was not inferior to radical hysterectomy plus postoperative adjuvant radiotherapy with respect to survival and was associated with fewer severe complications, findings that may also apply to earlier stages (IB–IIA) [8]. Nonetheless, dual-modality therapy has consistently been associated with higher toxicity in both PLWHIV and HIV-negative patients [9].

Despite the high burden of both HIV and cervical cancer in sub-Saharan Africa (SSA), to date, no study in this region has evaluated DFS and OS after trimodal therapy by HIV status for early stage cervical cancer. It is unclear whether current HIV management affects malignancy presentation and prognosis in the setting of intensive trimodal therapy. This study therefore aimed to characterize clinicopathological presentation, treatment-related morbidity, and survival outcomes of PLWHIV and HIV-negative patients with stage IA–IIA (FIGO 2018) cervical cancer who received trimodal treatment at a tertiary facility in Kenya.

## Methods

### Study setting

The study was conducted at the Chandaria Cancer and Chronic Care Centre (CCC) within MTRH, a tertiary hospital serving ~ 24 million people from western Kenya, eastern Uganda, and southern Sudan. CCC provides comprehensive gynecologic oncology services with a capacity for 10,000 patients and is equipped for cancer research and education [10].

### Study design, data source & patient identification

We conducted a descriptive retrospective cohort study using data from a prospectively maintained electronic REDCap database at the CCC. The study followed the Strengthening the Reporting of Observational Studies in Epidemiology (STROBE) checklist. We searched for eligible patients with cervical cancer diagnosis between January 1, 2014, and December 31, 2023. Cases identified were based on ICD-10 Diagnosis Code (C53 - Malignant neoplasm of cervix uteri) and procedure codes (radical hysterectomy (Type II/III) and bilateral pelvic lymphadenectomy as per the hospital’s procedure coding system). This search identified 275 patients. Each of these electronic medical records and surgical pathology reports were subsequently reviewed to confirm eligibility. Database records documented adjuvant therapy completion as binary (completed versus not initiated). Mid-treatment discontinuation was not systematically captured during the study period.

### Eligibility and study population

Inclusion criteria: women ≥ 18 years of age with histologically confirmed cervical cancer (FIGO 2018 stage IA–IIA) treated at MTRH during the study period, who completed trimodal therapy (type II/III radical hysterectomy with bilateral pelvic lymphadenectomy, followed by adjuvant CCRT, had documented intermediate- or high-risk pathology, and known HIV serology.

Exclusion criteria: low risk pathology not requiring adjuvant therapy, incomplete outcome data, simple hysterectomy, primary CCRT, or incomplete trimodal therapy (surgery, chemotherapy and radiotherapy).

Retrospective staging was done according to FIGO 2018 criteria using available surgical and pathological findings.

### Variables and definitions

Intermediate and high-risk categories were defined according to established Sedlis - Gynecologic Oncology Group (GOG-92) and Peters criteria [11–13].

“Intermediate risk: a tumor with positive lymphovascular space invasion (LVSI) combined with deep one-third stromal invasion, middle one-third stromal invasion plus tumor diameter ≥2 cm, superficial one-third stromal invasion plus tumor diameter ≥5 cm, or with no LVSI but with deep or middle one-third stromal invasion plus tumor diameter ≥4 cm.”

High-risk included positive pelvic lymph nodes, positive surgical margins, or parametrial involvement.

Treatment-related complications were graded using Radiation Therapy Oncology Group (RTOG) and Common Terminology Criteria for Adverse Events (CTCAE v3.0) (9, 10). Complications within four weeks of treatment were classified as early, and those occurring thereafter as late.

#### Survival endpoints

Disease-free survival (DFS) referred to the interval in months from hysterectomy to first recurrence, death, or last contact.

Overall survival (OS) referred to the interval in months from hysterectomy to death or last contact.

Treatment Protocol: Radical hysterectomy included resection of the uterus, parametrial tissue, and a margin of the upper vagina, consistent with type II/III Piver-Rutledge procedures. Standard adjuvant regimen comprised CCRT, including external beam radiation therapy (EBRT) of a total dose of 45–50.4 Gy (Gy) to the whole pelvis, delivered in daily fractions of 1.8–2 Gy, five days per week for 25 sessions. Dose escalation to 50.4 Gy was used if parametrial involvement or positive margins were present. Weekly cisplatin (40 mg/m²) was administered concurrently as a radiosensitizer; however it was held in the event of grade 3 or more neutropenia or creatinine > 1.5 mg/dL. Following EBRT completion, vaginal cuff brachytherapy was administered based on individualized assessment of surgical pathology and vaginal cuff anatomy.

Chemoradiation was administered per institutional practice to all patients receiving adjuvant therapy during the study period. This approach is consistent with Peters criteria for high-risk disease. For intermediate-risk patients, radiotherapy alone may suffice per Sedlis criteria (GOG-92); however, preoperative staging limitations in our setting necessitate heavy reliance on surgical pathology to guide adjuvant decisions. In this context, to avoid undertreatment of high-risk disease, chemoradiation was pragmatically administered to all adjuvant-eligible patients.

### Data collection and management

Data collection used a standardized, pre-piloted electronic data abstract form. The form covered demographic, pathological, clinical, and treatment outcome variables. To reduce selection bias, consecutive patients meeting eligibility criteria over 10 years were included. Incomplete or missing survival/recurrence information was supplemented via telephone contact with patients or caregivers as designated. Missing baseline and pathological characteristics data were tabulated separately. The data extraction form followed a validated framework (11).

### Statistical analysis

Sample size and power: No a priori power calculation was done. The study was hypothesis-generating due to operational constraints and anticipated low trimodal therapy completion rate in this setting. Loss to follow-up was addressed through active patient tracing (as described in data collection section). Last clinic visit/ treatment completion date was used as the censoring time in survival analyses when contact was unsuccessful.

Baseline clinicopathological features and treatment-related toxicities were summarised descriptively and stratified by HIV status. Continuous variables were compared using Student’s t test or Mann–Whitney U test, and categorical variables with chi-square or Fisher’s exact test- not to test hypotheses, but to characterize patterns.

Survival (DFS and OS) was estimated using Kaplan–Meier methods, curves were plotted with 95% confidence intervals and compared by log-rank tests with exploratory intent. Complete-case approach was employed for survival analyses. Multivariable Cox proportional hazards models adjusted for age, FIGO stage, and LVSI; interaction terms were evaluated where appropriate. Two-tailed *p* < 0.05 was considered statistically significant. Subgroup and interaction analyses were conducted post hoc and were exploratory. Analyses were performed in SPSS version 23.0 (IBM Corp., Armonk, NY, USA).

### Ethical considerations and consent to participate

The study received ethical approval from the Institutional Research and Ethics Committee (IREC) of Moi University/MTRH (Approval No. 0004931) and was licensed by the National Commission for Science, Technology, and Innovation (NACOSTI) (License No. NACOSTI/P/24/414426). The IREC granted a waiver of informed consent for this study, as it involved secondary analysis of anonymized, routinely collected clinical data with minimal risk to participants. Permission to access patient records was also obtained from MTRH management (Ref: ELD/MTRH/R&P/10/2/V.2/2010).

Data confidentiality was safeguarded in accordance with the Declaration of Helsinki. All patient identifiers were anonymized prior to analysis and reporting.

## Results

**Fig. 1:**
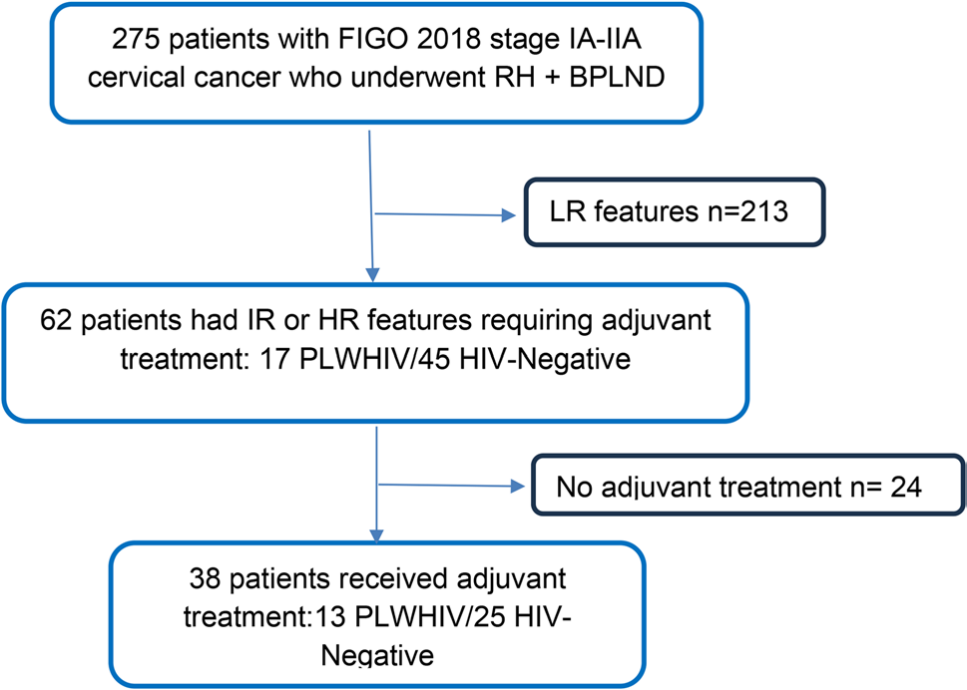
Data screening process. RH: Radical Hysterectomy, BPLND: Bilateral pelvic lymph node dissection, LR: Low-Risk, IR: Intermediate-Risk, HR: High-Risk, PLWHIV: People Living with HIV

Over the 10-year study period, 275 patients with cervical cancer underwent radical hysterectomy with bilateral pelvic lymphadenectomy at MTRH. Of 62 patients meeting criteria for adjuvant therapy (17 PLWHIV, 45 HIV-negative), 38 (61.3%) completed trimodal therapy. This corresponded to a completion rate of 76.5% (13/17) among PLWHIV versus 55.6% (25/45) among HIV-negative patients (Fig. 1).

The clinicopathological characteristics of eligible participants are summarised in Table 1. The majority of patients were over 40 years of age, with comparable median ages (48 vs. 49 years; *p* = 0.29). Most (92.1%) presented with Eastern Cooperative Oncology Group (ECOG) performance status 0. CD4 counts were available for only 23.1% of PLWHIV (of 17 patients eligible for adjuvant therapy), with a mean of 486 cells/mm³ (SD 146). Most patients presented with stage IB2 (39.5%) or IB3 (34.2%) disease. Squamous cell carcinoma predominated (92.1%), accounting for all cases in PLWHIV and 88.0% of HIV-negative cases (*p* = 0.69). Median tumour size was 4.0 cm in both groups. Positive pelvic lymph nodes were the leading high-risk feature, affecting 53.8% of PLWHIV versus 40.0% of HIV-negative. LVSI was observed in half of cases, 69.2% of PLWHIV and 40.0% of HIV-negative patients (*p* = 0.23). Data on deep stromal invasion were missing in 55.3% of the cohort, precluding precise risk stratification for the cohort (Table 1).

**Table 1:** Baseline characteristics in early-stage cervical cancer by HIV Status (*N* = 38)

Characteristics		PLWHIV (*n* = 13)	HIV-negative (*n* = 25)	Total (*n* = 38)	*p*-value
Demographic & Clinical					
Median age, years (IQR)	48 (44–52)		49 (41–55)	48.5 (42–54)	0.29
ECOG 0, n (%)	12 (92.3%)		23 (92.0%)	35 (92.1%)	1.00
FIGO 2018 stage, n (%)					0.37
IB1	2 (15.4%)		4 (16.0%)	6 (15.8%)	
IB2	5 (38.5%)		10 (40.0%)	15 (39.5%)	
IB3	3 (23.1%)		10 (40.0%)	13 (34.2%)	
IIA	3 (23.1%)		1 (4.0%)	4 (10.5%)	
Histology, n (%)					0.69
Squamous cell	13 (100%)		22 (88.0%)	35 (92.1%)	
Adenocarcinoma	0		2 (8.0%)	2 (5.3%)	
Adeno-squamous	0		1 (4.0%)	1 (2.6%)	
Median tumor size, cm77.5 (IQR)	4.0 (3.0–5.0)		4.0 (2.8-5.0)	4.0 (2.9-5.0)	0.31
Pathological risk features, n (%)					
Lymphovascular space invasion (LVSI)					0.23
Present	9 (69.2%)		10 (40.0%)	19 (50.0%)	
Absent	4 (30.8%)		15 (60.0%)	19 (50.0%)	
Deep stromal invasion					1.00
Present	5 (38.5%)		8 (32.0%)	13 (34.2%)	
Absent	1 (7.7%)		3 (12.0%)	4 (10.5%)	
Missing	7 (53.8%)		14 (56.0%)	21 (55.3%)	
Parametrial involvement					1.00
Present	3 (23.1%)		7 (28.0%)	10 (26.3%)	
Absent	7 (53.8%)		12 (48.0%)	19 (50.0%)	
Missing	3 (23.1%)		6 (24.0%)	9 (23.7%)	
Pelvic lymph nodes					
Positive	7 (53.8%)		10 (40.0%)	17 (44.7%)	0.40
Negative	5 (38.5%)		13 (52.0%)	18 (47.4%)	
Missing	1 (7.7%)		2 (8.0%)	3 (7.9%)	
Surgical margins					1.00
Positive	2 (15.4%)		3 (12.0%)	5 (13.2%)	
Negative	9 (69.2%)		17 (68.0%)	26 (68.4%)	
Missing	2 (15.4%)		5 (20.0%)	7 (18.4%)	

*Percentages may not total 100 because of rounding

*SD* Denotes standard deviation

The clinicopathological characteristics of all 62 patients meeting criteria for adjuvant therapy are summarised in supplementary Table 1.

The time from surgery to the initiation of adjuvant CCRT was a median of 77.5 days (interquartile range [IQR], 42 to 210). PLWHIV started slightly earlier (median, 72 days) compared to HIV-negative patients (median, 81 days). Treatment time was 63 days among PLWHIV and 73 days among HIV-negative patients.

**Fig. 2:**
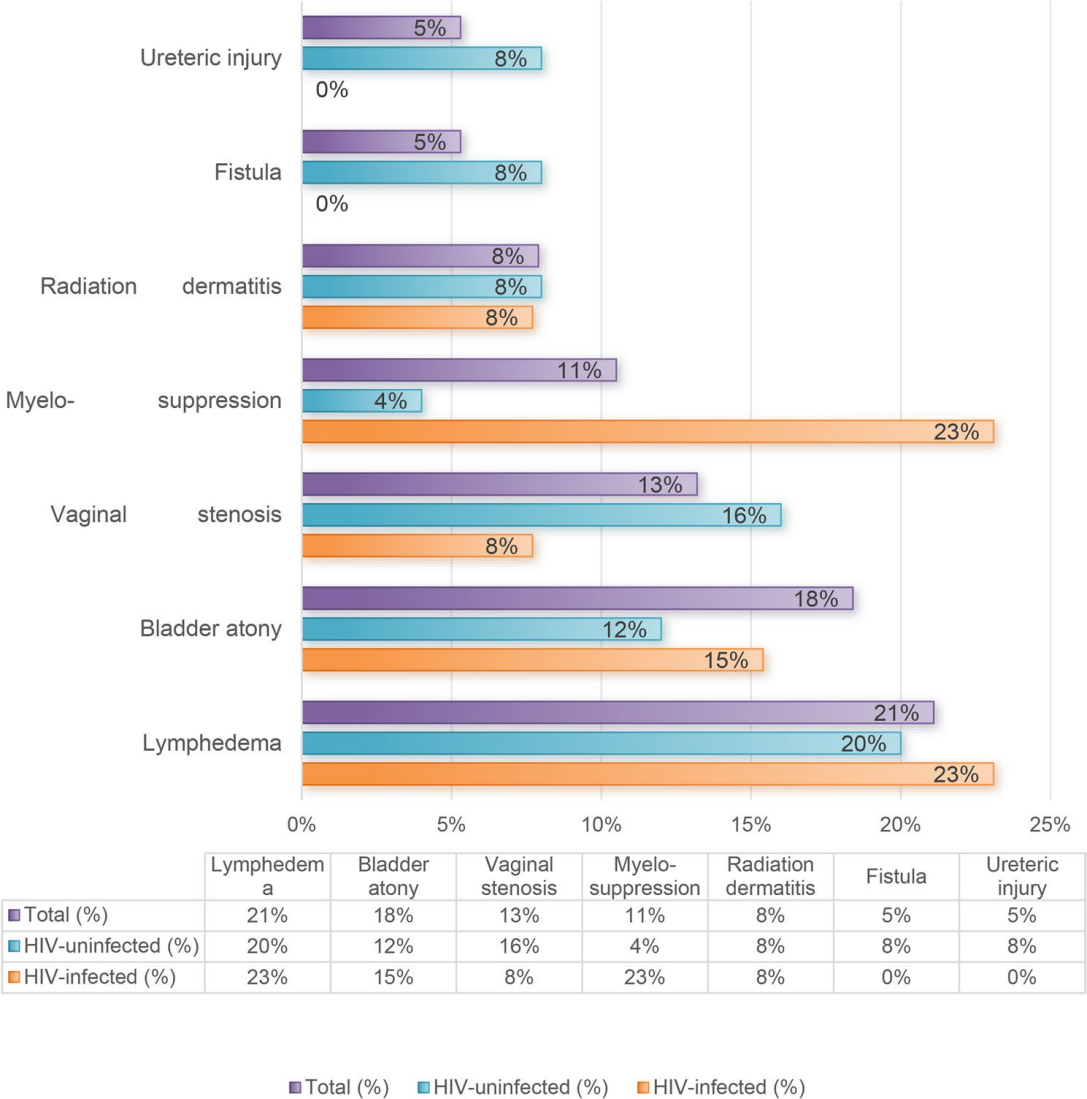
Treatment related complications in early-stage cervical cancer by HIV status (*N* = 38)

Overall, lymphedema was the most frequently reported any-grade chronic toxicity (21.1%), followed by bladder dysfunction. Myelosuppression was recorded in 3 of 13 PLWHIV (23.1%) and 1 of 25 HIV-negative patients (4.0%). Ureteric injury and fistula formation were reported only in HIV-negative patients (8.0% each)(Fig. 2).

**Fig. 3:**
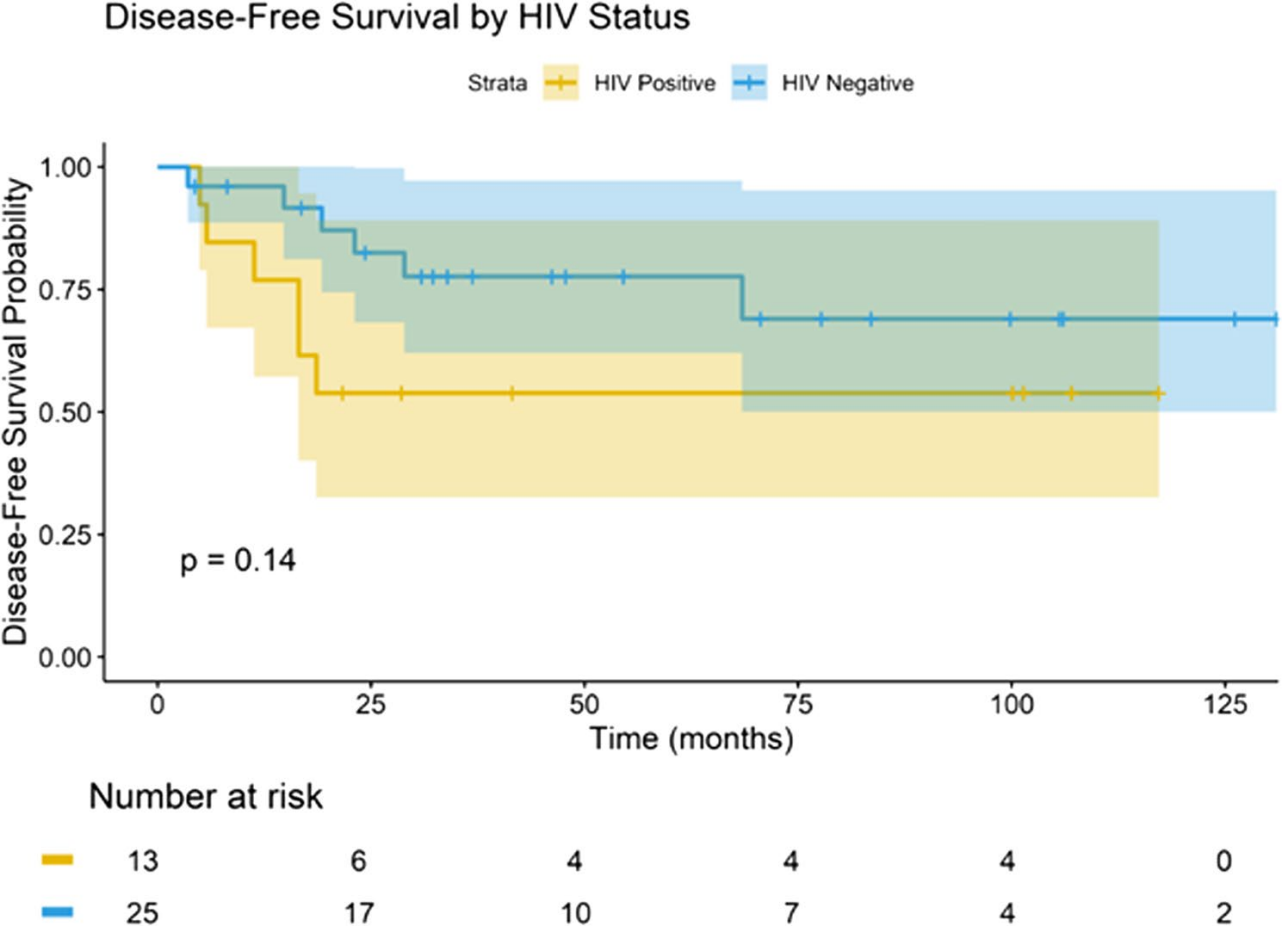
Kaplan-Meier Curve for DFS by HIV status

**Fig. 4:**
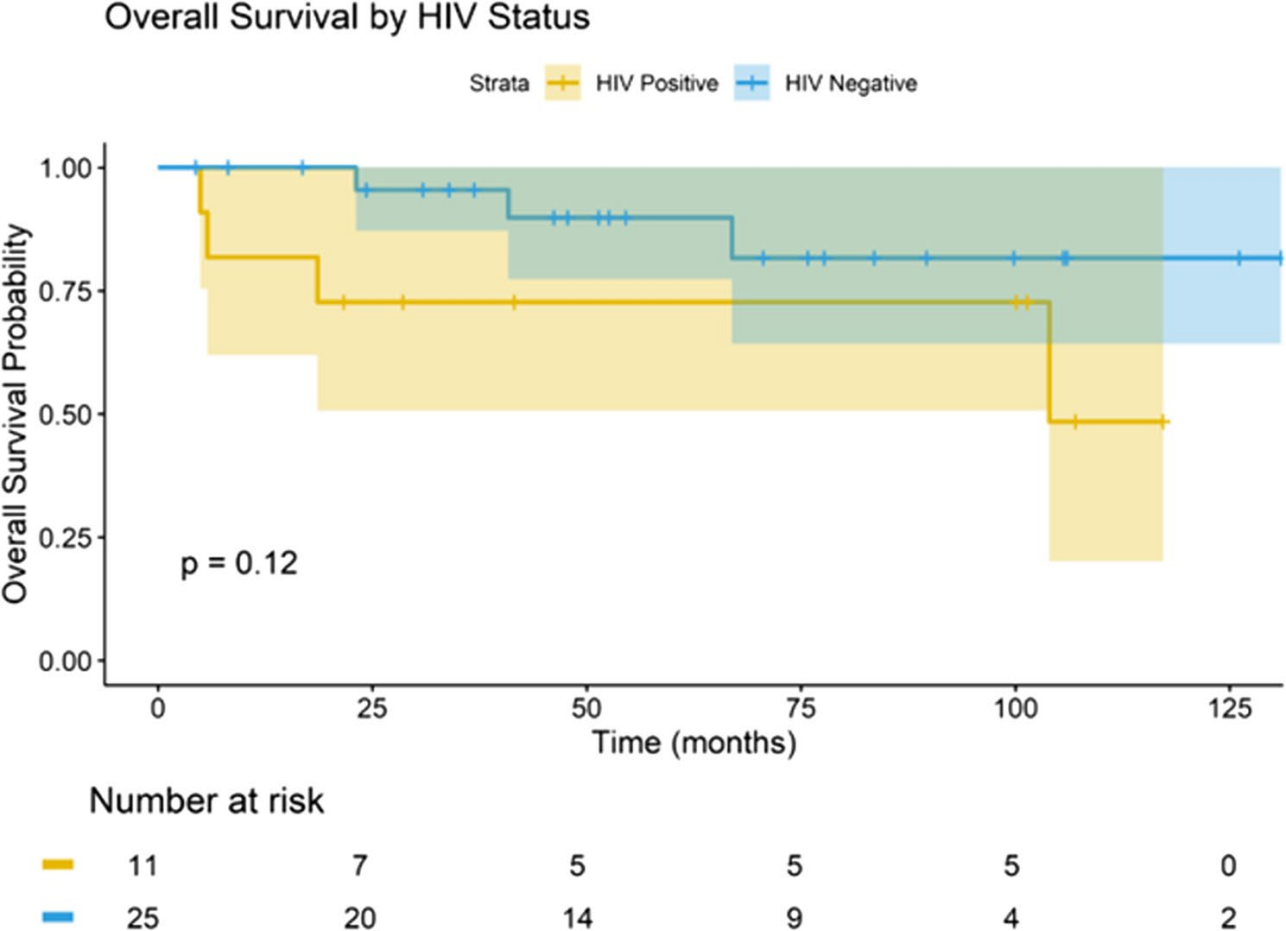
Kaplan-Meier Curve for Overall Survival by HIV status

The median potential follow-up estimated was 48.2 months (95% CI 36.1–60.3). The median DFS was 10.6 months among PLWHIV and 15.2 months (95% CI, 10.1–20.3) in HIV-negative women (log-rank *p* = 0.14). The estimated three-year DFS was 53.8% in the PLWHIV group versus 77.6% in the HIV-negative group. At five years, DFS rate was 69.0% in HIV-negative patients but could not be estimated for PLWHIV due to a small number of patients remaining at risk (Fig. 3). Median OS was 14.5 months in the PLWHIV group and 21.1 months in the HIV-negative group (log-rank *p* = 0.12). At five years, OS was 48.5% in HIV-negative women but not estimable in the PLWHIV cohort. Within 30 days of completing trimodal therapy, two PLWHIV experienced early mortality (one from sepsis, one from pulmonary embolism), and their event times were not captured in initial survival coding. After audit, both were included as events at time zero (Day 0 post-hysterectomy), resulting in *n* = 13 in analyses but only 11 at-risk at Day 60, which is why the Kaplan-Meier curve starts at 11 (Fig. 4).

Adjusted Cox regression (adjusted for age, FIGO stage, and lymphovascular space invasion) yielded a hazard ratio (aHR) for death of 0.29 (95% CI, 0.06–1.49; *P* = 0.14) for HIV-negative relative to PLWHIV patients.

## Discussion

This retrospective cohort study from western Kenya provides a real-world assessment of treatment outcomes of trimodal therapy for early-stage cervical cancer, with attention to HIV related patterns.

Our principal finding is that among the small cohort of women who completed this intensive regimen, despite comparable clinicopathological profiles at baseline and near-identical treatment delivery; PLWHIV experienced higher rates of treatment-related morbidity (especially myelosuppression) and a trend (though statistically nonsignificant) toward earlier disease recurrence. There is room to challenge assumptions that ART-normalized immunity fully mitigates oncologic disparities.

A marked finding was the fewer than two-thirds (61%) completion rate of adjuvant therapy. Treatment uptake did not differ by HIV status, suggesting that existing barriers are systemic rather than disease-specific. These results are consistent with broader evidence; Putri et al., in a systematic review on global cancer management observed low adherence to treatment guidelines ranging 42%–54% [14]. Challenges in delivering complete, guideline-adherent care are not confined to our setting.

The median duration of 77.5 days (11 weeks) exceeded the 6-week benchmark associated with optimal survival [15, 16]. Among PLWHIV, a modestly shorter interval to adjuvant therapy initiation (median 72 versus 81 days) and therapy completion (63 versus 73 days) was observed compared to HIV-negative patients. This may be due to system-level advantages conferred by the institutions’ HIV care infrastructure. At the study site, PLWHIV are typically enrolled in the Academic Model Providing Access to Healthcare (AMPATH) programme. The AMPATH programme entails a structured HIV care system involving longitudinal clinical follow-up, active patient tracking and community health worker engagement [17]. This may have facilitated slightly more consistent clinic engagement and navigation through to radiation oncology pathways. HIV-negative patients lack an equivalent care coordination, which may also explain higher trimodal therapy completion rate observed among PLWHIV (76.5% versus 55.6%). These findings highlight opportunities for system level improvement. Interventions could include: (a) pre-operative radiation oncology consultations to schedule radiotherapy prior to pathology results; (b) centralized coordination of care with a dedicated surgical-to-radiation pathway with defined 2-week referral targets; (c) adapting AMPATH’s patient tracking infrastructure particularly centralized appointment coordination and community health worker engagement for cervical cancer patients regardless of HIV status. These could represent high impact target areas, potentially reducing delay to adjuvant therapy initiation.

You et al. in a study in China provided strong corroboration that in stage IB2–IIA disease, postponing adjuvant therapy for greater than 5 weeks from surgery independently reduced survival, mainly through increased local recurrence [18]. National radiotherapy shortages, limited radiotherapy capacity, treatment delays, high out-of-pocket costs, and therapy interruption due to machine breakdowns, are common in LMICs. The national radiotherapy coverage in Kenya remains under 50%, with fewer than 20 functional machines for the over 50 million population [19]. The data points to a reality that even when curative-intent therapy is indicated and initiated, delivery gaps substantially narrow the window for cure. Enhancing survival outcomes for this cohort of women in Kenya therefore requires a dual focus: addressing systemic barriers that impede access to standard care and advancing treatment options.

One-third of patients were PLWHIV, consistent with Kenyan and regional series but lower than Southern Africa, where prevalence among women with cervical cancer reaches 52–75% [3, 20–22]. Median age at treatment was similar between groups and most women presented with preserved functional status (ECOG 0 in 92.1%), comparable to Zambian cohorts, indicating careful preoperative selection and suitability for curative multimodal therapy despite the dual burden of HIV and cancer [23]. Immunovirological data were largely absent, much like South African cohorts [24], reflecting the gaps in integration between oncology and HIV care, inconsistent documentation of cancer treatment records and limited laboratory capacity in most SSA settings.

Survival was favorable at one year but diverged within three, with PLWHIV experiencing earlier recurrence and non-estimable five-year survival. In comparison with literature, the 5-year OS of 69% in HIV-negative patients falls below the 78–90% rates reported in pivotal trials for stage IB-IIA disease with high-risk features treated with adjuvant chemoradiation [25]. The numerically inferior survival outcomes for PLWHIV align with studies from Uganda and Brazil [26, 27]. Evidence from a Botswanan study found that under robust, protocolized care, survival disparities by HIV status were eliminated [28]. In settings without such standardized support, our findings suggest HIV may be a marker for increased susceptibility to worse survival outcomes. Improving access alone may not be sufficient to eliminate survival disparities, especially if biological vulnerabilities persist. Evidence suggests that even virologically suppressed PLWHIV may harbor persistent immune dysfunction (PD-1/PD-L1 expression, T-cell exhaustion) [29] which may explain study outcome disparities. The study’s limited CD4/viral load data (present for only 23.5% of PLWHIV) however, preclude definitive assessment.

The incidence of lymphedema in the study was 21.1%, higher than rates reported in other retrospective analyses in India (15%) and Japan (9.5%) [30, 31]. This may be explained by a number of factors in our setting including, routine extended lymph node dissection and limited use of modern surgical techniques such as sentinel node mapping or nerve-sparing during hysterectomy. All patients received adjuvant pelvic radiotherapy, and there is emerging evidence that points to radiation being an independent predictor of lymphedema. Hu et al. [32], in a meta-analysis, and Chang et al. [33], in a propensity score - matched study each demonstrated a threefold excess risk. Radiation-induced fibrosis and damage to lymphatic channels and nodes left intact after surgery likely disrupt drainage. In our context, improved lymphedema management services (compression garments, physiotherapy) are critical. These complications often translate into reduced quality of life and long-term disability.

A trend toward greater myelosuppression in PLWHIV (23.1% vs. 4.0%) was observed. This accords with evidence that antiretrovirals may interact with chemotherapy, especially cisplatin, amplifying hematologic toxicity [34]. Proactive management strategies, including closer monitoring and growth factor support (not routinely available) are required. Curiously, ureteric injury and fistula occurred only in HIV-negative patients. This pattern is likely multifactorial. HIV-negative patients may have had more extensive surgical dissection driven by bulkier tumors and higher rates of parametrial involvement; consequently, increasing ureteric injury risk. Enhanced intraoperative vigilance during surgery in relation to PLWHIV (concerns regarding tissue fragility, bleeding, or infection risk) may have mitigated such injuries. Although causality cannot be inferred, the clustering of ureteric injury and fistula formation may reflect delayed intraoperative recognition or suboptimal early urological intervention,

### Strengths and limitations

This is the first study from a low-resource setting to report outcomes of trimodal therapy stratified by HIV status in early-stage cervical cancer. Use of a prospective database for identification and application of standardized risk criteria, provided a realistic snapshot of cancer care delivery in Kenya. The extended study period enabled robust description of real-world patterns of care and survival outcomes, providing important context for oncology practice in sub-Saharan Africa.

Nonetheless, our findings should be interpreted in the context of the study’s limitations. Its small sample size and retrospective nature precluding definitive conclusions and limits causal inference. A post-hoc power analysis indicated that the study had a 26% power to detect an HR of 0.30 at α = 0.05, highlighting the need for larger studies. Missing CD4 counts and viral load for the majority of PLWHIV participants prevents assessment of immunovirological correlates of outcomes.

Absent deep stromal invasion data (55.3%) may have also misclassified intermediate-risk status (per Sedlis criteria). Multiple imputation was deemed inappropriate because data were not missing at random (for example, CD4 testing was inconsistent before 2018). A complete-case sensitivity analysis (*n* = 17 with full pathological data) was done, which reproduced the direction of effect (aHR 0.25, *p* = 0.21). This supports the robustness of our findings despite missingness.

Source clinical database documented adjuvant therapy status as binary categories only (completed versus not initiated). Mid-treatment discontinuation was not systematically captured during the ten-year period (2014–2023). All reported complications therefore reflect only the 38 patients who completed therapy, potentially underestimating true treatment-related morbidity. The 38.7% non-initiation rate (24 of 62 eligible patients) represents a critical gap in curative-intent care delivery. The reasons for non-initiation were also not systematically documented, precluding assessment of barriers.

Dosimetric and cycle level analysis could also not be done as treatment delivery metrics were inconsistently recorded even among completers. These documentation gaps reflect real-world constraints, especially in the Global South and offer an opportunity for clinical data systems improvement for future prospective work.

## Conclusion

PLWHIV receiving trimodal therapy for early-stage cervical cancer showed trends toward greater toxicity and poorer survival, particularly within the first two years. Although underpowered for statistical significance, the trends were consistent. Larger, prospective multicentre studies integrating systematic HIV monitoring and standardized treatment protocols are needed to clarify biological interactions, optimize outcomes, and reduce disparities in low-resource settings.

## Supplementary Information

Supplementary Material 1.

Supplementary Material 2.

## Data Availability

The de-identified datasets used for this study are available from the corresponding author upon reasonable request.

## Abbreviations

AIDS: Acquired Immunodeficiency Syndrome
AMPATH: Academic Model Providing Access to Healthcare
ART: Antiretroviral Therapy
BPLND: Bilateral Pelvic Lymph Node Dissection
CCRT: Concurrent Chemoradiotherapy
CD4: Cluster of Differentiation 4
DFS: Disease-Free Survival
EBRT: External Beam Radiation Therapy
HIV: Human Immunodeficiency Virus
IR: Intermediate-Risk
LR: Low-Risk
LVSI: Lymphovascular Space Invasion
MTRH: Moi Teaching and Referral Hospital
OS: Overall Survival
PLWHIV: People Living with HIV
REDCap: Research Electronic Data Capture
