# Supplementary material for: Characterizing trimodal therapy outcomes by HIV status in early-stage cervical cancer: a retrospective cohort study from a Kenyan tertiary centre
Source: BMC Cancer. 2026 Mar 9;26:488. doi: 10.1186/s12885-026-15761-5 (PMC13088384; doi:10.1186/s12885-026-15761-5)
Supplement: Supplementary file 1 — Supplementary Material 1. [file 12885_2026_15761_MOESM1_ESM.docx]

**STROBE Statement—checklist of items that should be included in reports of observational studies**

|  | Item No. | Recommendation | Page  No. | Relevant text from manuscript |
| --- | --- | --- | --- | --- |
| **Title and abstract** | 1 | (*a*) Indicate the study’s design with a commonly used term in the title or the abstract | Title (p.1) | A Retrospective Cohort Study. |
|  |  | (*b*) Provide in the abstract an informative and balanced summary of what was done and what was found | Abstract (p.2-4) | Descriptive retrospective cohort, specifies trimodal regimen, and reports 3-year DFS/OS, HR, and toxicity. |
| Introduction | | | |  |
| Background/rationale | 2 | Explain the scientific background and rationale for the investigation being reported | Intro (p.5-6) | To date, no study in this region has evaluated DFS and OS after trimodal therapy by HIV status. |
| Objectives | 3 | State specific objectives, including any prespecified hypotheses | End of Intro (p.6) | Characterize clinicopathological presentation, treatment-related morbidity, and survival outcomes… stratified by HIV status. |
| Methods | | | |  |
| Study design | 4 | Present key elements of study design early in the paper | Methods (p.6-8) | descriptive retrospective cohort*, 2014–2023, single-center. |
| Setting | 5 | Describe the setting, locations, and relevant dates, including periods of recruitment, exposure, follow-up, and data collection | Methods (p.6-8) | MTRH/CCC, Eldoret; appx 24M catchment; Jan 2014–Dec 2023. |
| Participants | 6 | (*a*) *Cohort study*—Give the eligibility criteria, and the sources and methods of selection of participants. Describe methods of follow-up  *Case-control study*—Give the eligibility criteria, and the sources and methods of case ascertainment and control selection. Give the rationale for the choice of cases and controls  *Cross-sectional study*—Give the eligibility criteria, and the sources and methods of selection of participants | Methods (p.6-8) | Inclusion/exclusion defined; identification via REDCap + ICD-10/procedure codes (n=275 to n= 38). |
|  |  | (*b*) *Cohort study*—For matched studies, give matching criteria and number of exposed and unexposed  *Case-control study*—For matched studies, give matching criteria and the number of controls per case |  |  |
| Variables | 7 | Clearly define all outcomes, exposures, predictors, potential confounders, and effect modifiers. Give diagnostic criteria, if applicable | Methods (p.8-9)  Variables & Treatment Protocol | HIV status (exposure); DFS/OS/toxicity (outcomes); age/stage/LVSI (confounders). Definitions (i.e, Sedlis/Peters) cited. |
| Data sources/ measurement | 8* | For each variable of interest, give sources of data and details of methods of assessment (measurement). Describe comparability of assessment methods if there is more than one group | Methods (p.9) Variables & Data collection | REDCap, pathology reports. Toxicity graded per RTOG/CTCAE. |
| Bias | 9 | Describe any efforts to address potential sources of bias | Methods (p.9) Data collection | To reduce selection bias, consecutive patients meeting eligibility criteria over 10 years were included…. |
| Study size | 10 | Explain how the study size was arrived at | Methods (p.9-10) Statistical analysis | No a priori power calculation… hypothesis-generating… post-hoc power = 26% to detect HR=0.30. |

Continued on next page

| Quantitative variables | 11 | Explain how quantitative variables were handled in the analyses. If applicable, describe which groupings were chosen and why | Methods (p.9-10) Statistical analysis | Continuous variables summarized as median (IQR) or mean (SD); compared with Mann–Whitney or t-test. |
| --- | --- | --- | --- | --- |
| Statistical methods | 12 | (*a*) Describe all statistical methods, including those used to control for confounding | Methods (p.9-10) Statistical analysis | KM/log-rank + Cox (aHR adjusted for age/stage/LVSI). SPSS v23 |
|  |  | (*b*) Describe any methods used to examine subgroups and interactions | Methods (p.9-10) Statistical analysis | Subgroup and interaction analyses were conducted post hoc and were exploratory. |
|  |  | (*c*) Explain how missing data were addressed | Methods (p.9-10) Data collection & Statistical analysis | Missing baseline and pathological characteristics data were tabulated separately. complete-case + sensitivity analysis for missing data; |
|  |  | (*d*) *Cohort study*—If applicable, explain how loss to follow-up was addressed  *Case-control study*—If applicable, explain how matching of cases and controls was addressed  *Cross-sectional study*—If applicable, describe analytical methods taking account of sampling strategy | Methods (p.9-10) Data collection & Statistical analysis | Incomplete or missing survival/recurrence information were supplemented via telephone contact with patients or caregivers as designated. Last clinic visit/ treatment completion date was used as the censoring time in survival analyses when contact was unsuccessful. |
|  |  | (*e*) Describe any sensitivity analyses | NA | No formal multiplicity adjustment were applied because all analyses were exploratory |
| Results | | | | |
| Participants | 13* | (a) Report numbers of individuals at each stage of study—eg numbers potentially eligible, examined for eligibility, confirmed eligible, included in the study, completing follow-up, and analysed | Fig 1 and Results, p.11-12 | Flow diagram (275 → 213 LR → 62 IR/HR → 38 completed). Exclusions: no adjuvant (n=24) |
|  |  | (b) Give reasons for non-participation at each stage |  |  |
|  |  | (c) Consider use of a flow diagram |  |  |
| Descriptive data | 14* | (a) Give characteristics of study participants (eg demographic, clinical, social) and information on exposures and potential confounders | Table 1, (p.11-12) | Table 1 (Baseline characteristics, “Missing” rows for pathology variables)  Methods: Variables & Statistical analysis (p-value thresholds, covariates in model)  Results: Pathological risk features, CD4 availability  Discussion: Limitations section details rationale for no imputation. |
|  |  | (b) Indicate number of participants with missing data for each variable of interest |  |  |
|  |  | (c) *Cohort study*—Summarise follow-up time (eg, average and total amount) |  |  |
| Outcome data | 15* | *Cohort study*—Report numbers of outcome events or summary measures over time | Results, p.11-12, 23-24 | Results (DFS/OS medians, 3- and 5-year estimates). Figures 3–4 (KM curves with 95% CI bands and number-at-risk table) |
|  |  | *Case-control study—*Report numbers in each exposure category, or summary measures of exposure |  |  |
|  |  | *Cross-sectional study—*Report numbers of outcome events or summary measures |  |  |
| Main results | 16 | (*a*) Give unadjusted estimates and, if applicable, confounder-adjusted estimates and their precision (eg, 95% confidence interval). Make clear which confounders were adjusted for and why they were included | Results, p.11-12 | Results (aHR 0.29, CI, p-value)  Methods (Statistical analysis: “adjusted for age, FIGO stage, and LVSI”)  Discussion (interpretation of HR magnitude despite non-significance) |
|  |  | (*b*) Report category boundaries when continuous variables were categorized | NA | Age was analyzed continuously but also dichotomized at <50 vs. ≥50 years for subgroup exploration (no formal cutoff pre-specified |
|  |  | (*c*) If relevant, consider translating estimates of relative risk into absolute risk for a meaningful time period | NA |  |

Continued on next page

| Other analyses | 17 | Report other analyses done—eg analyses of subgroups and interactions, and sensitivity analyses | Methods (Statistical analysis, p.9-10) & Results (p.11-12) & Discussion (p.12-15) | A post hoc sensitivity analysis using a complete-case subset (n = 17) with fully documented deep stromal invasion reproduced the direction of the survival effect (aHR 0.25, 95% CI 0.04–1.58; p = 0.21  No formal subgroup analyses (e.g., by CD4, ART regimen) were feasible due to missing immunovirological data. All additional analyses were exploratory. |
| --- | --- | --- | --- | --- |
| Discussion | | | | |
| Key results | 18 | Summarise key results with reference to study objectives | Abstract (p.2-4)  Results (p.11-12) & Discussion (p.12-15) | 38 women completing trimodal therapy (13 HIV-positive, 25 HIV-negative), baseline characteristics were comparable. However, HIV-positive patients experienced higher myelosuppression (23.1% vs. 4.0%) and reduced 3-year DFS (53.8% vs. 77.6%) and median OS (14.5 vs. 21.1 months). Adjusted Cox regression showed a 71% lower risk of death in HIV-negative patients (aHR 0.29, 95% CI 0.06–1.49; p = 0.14). |
| Limitations | 19 | Discuss limitations of the study, taking into account sources of potential bias or imprecision. Discuss both direction and magnitude of any potential bias | Discussion (p.12-16)  Strengths and limitations | Key limitations include small sample size (post-hoc power = 26% to detect HR = 0.30), retrospective design (limiting causal inference), and high missingness in CD4 counts (76.9%) and deep stromal invasion (55.3%). This precludes adjustment for immunovirological status and may misclassify intermediate-risk patients. Although data were not missing at random, sensitivity analysis supports robustness of findings. |
| Interpretation | 20 | Give a cautious overall interpretation of results considering objectives, limitations, multiplicity of analyses, results from similar studies, and other relevant evidence |  | PLHIV may remain vulnerable to poorer trimodal therapy outcomes, not necessarily due to biological differences alone, but as a marker of systemic inequity. The non-significant HR (0.29) aligns with regional studies from Uganda and Brazil, while contrast with Botswana implies that protocolized, integrated HIV–oncology care may eliminate disparities. Caution is warranted given limited power; findings are hypothesis-generating, not confirmatory. |
| Generalisability | 21 | Discuss the generalisability (external validity) of the study results | Intro (p.5-6) & Discussion (p.12-16) | Results likely generalize to similar LMIC settings in sub-Saharan Africa. Findings may not apply to high-income settings with protocolized HIV–oncology integration. |
| Other information | |  | | |
| Funding | 22 | Give the source of funding and the role of the funders for the present study and, if applicable, for the original study on which the present article is based | P.16 | This work was supported by the U54 program (Grant ID: EACHC-444). The funder had no role in study design; collection, analysis, and interpretation of data; writing of the article; nor the decision to submit the report for publication. |

*Give information separately for cases and controls in case-control studies and, if applicable, for exposed and unexposed groups in cohort and cross-sectional studies.

**Note:** An Explanation and Elaboration article discusses each checklist item and gives methodological background and published examples of transparent reporting. The STROBE checklist is best used in conjunction with this article (freely available on the Web sites of PLoS Medicine at http://www.plosmedicine.org/, Annals of Internal Medicine at http://www.annals.org/, and Epidemiology at http://www.epidem.com/). Information on the STROBE Initiative is available at www.strobe-statement.org.
