# Supplementary material for: Characterizing trimodal therapy outcomes by HIV status in early-stage cervical cancer: a retrospective cohort study from a Kenyan tertiary centre
Source: BMC Cancer. 2026 Mar 9;26:488. doi: 10.1186/s12885-026-15761-5 (PMC13088384; doi:10.1186/s12885-026-15761-5)
Supplement: Supplementary file 2 — Supplementary Material 2. [file 12885_2026_15761_MOESM2_ESM.pdf]

Supplementary Table 1: Treatment Delivery Metrics and Outcomes by HIV Status Among Patients Meeting Criteria for Adjuvant Therapy (N=62).

Variable	PLWHIV (N=17)	HIV-Negative (N=45)	Total (N=62)	p-value
**Treatment Completion**				
Completed trimodal therapy	13 (76.5%)	25 (55.6%)	38 (61.3%)	0.14
No adjuvant therapy initiated	4 (23.5%)	20 (44.4%)	24 (38.7%)	
**Timing Metrics**				
Days from surgery to CCRT initiation				
Median [IQR]	72 [42–180]	81 [45–210]	77.5 [42–210]	0.68
Missing data	4 (23.5%)	20 (44.4%)	24 (38.7%)	
**Days from CCRT initiation to completion**				
Median [IQR]	63 [35–84]	73 [32–189]	70.5 [32–189]	0.21
Missing data	7 (41.2%)	19 (42.2%)	26 (41.9%)	
**Risk Features**				
High-risk features (Peters criteria)				
Positive pelvic nodes	10 (58.8%)	25 (55.6%)	35 (56.5%)	0.82
Parametrial involvement	4 (23.5%)	10 (22.2%)	14 (22.6%)	0.91
Positive surgical margins	2 (11.8%)	6 (13.3%)	8 (12.9%)	0.87
Intermediate-risk features				
LVSI present	11 (64.7%)	18 (40.0%)	29 (46.8%)	0.09
Deep stromal invasion documented	7 (41.2%)	13 (28.9%)	20 (32.3%)	0.37
Deep stromal invasion missing†	9 (52.9%)	28 (62.2%)	37 (59.7%)	

Complication	Toxicity Grade*	PLWHIV (n=13)	HIV- Negative (n=25)	Total (n=38)
**Intraoperative events**				
Ureteric injury	Grade 3 (CTCAE)	0 (0%)	2 (8.0%)	2 (5.3%)
**Acute toxicities**				
Radiation dermatitis	Any grade (RTOG)	1 (7.7%)	2 (8.0%)	3 (7.9%)
Myelosuppression	Grade ≥3 (CTCAE)	3 (23.1%)	1 (4.0%)	4 (10.5%)
**Chronic toxicities**				
Bladder dysfunction/atony	Any grade (CTCAE)	3 (23.1%)	4 (16.0%)	7 (18.4%)
Lymphedema	Any grade (CTCAE)	3 (23.1%)	5 (20.0%)	8 (21.1%)
Vaginal stenosis	Any grade (RTOG)	1 (7.7%)	5 (20.0%)	6 (15.8%)
Fistula formation	Grade 3 (CTCAE)	0 (0%)	2 (8.0%)	2 (5.3%)

[1] *Abbreviations: PLWHIV, People Living with HIV; CTCAE, Common Terminology Criteria for Adverse Events; RTOG, Radiation Therapy Oncology Group.*
[2] *Values are presented as numbers and percentages, n (%).*

[3] *Complications were classified as acute (occurring ≤4 weeks after treatment completion) or chronic (>4 weeks). Radiation dermatitis was uniformly acute; lymphedema, bladder dysfunction, and vaginal stenosis were uniformly late toxicities.*
**Grade documentation limitations:*
Grade 3 ureteric injury/fistula based on requirement for surgical intervention (ureteric stenting/reimplantation or fistula repair), consistent with CTCAE Grade 3 definition.
Grade ≥3 myelosuppression based on protocol criteria requiring cisplatin hold for grade ≥3 neutropenia/thrombocytopenia.
Any-grade reporting for lymphedema, bladder dysfunction, vaginal stenosis, and radiation dermatitis due to inconsistent grade-level documentation in source database.
